# The perception of territory and personal space invasion among hospitalized patients

**DOI:** 10.1371/journal.pone.0198989

**Published:** 2018-06-13

**Authors:** Caroline Roveri Marin, Renata Cristina Gasparino, Ana Claudia Puggina

**Affiliations:** 1 Department of Collective Health, Faculty of Medicine Jundiaí, Jundiaí, São Paulo, Brazil; 2 School of Nursing, University of Campinas, Campinas, São Paulo, Brazil; 3 Post-graduate Program in Nursing, Guarulhos University, Guarulhos, São Paulo, Brazil; National Yang-Ming University, TAIWAN

## Abstract

**Objectives:**

1) To identify the patient’s perception of invasion of territorial and personal space and 2) to evaluate whether personal characteristics, housing conditions and characteristics of the hospital unit affect this perception.

**Methods:**

Analytical, cross-sectional and quantitative study. An adapted version of the “Anxiety Due to Territory and Space Intrusion Questionnaire” was applied with patients hospitalized in the internal medicine and maternity wards and in the ward for patients with private health insurance of a university hospital in the state of São Paulo.

**Results:**

The sample consisted of 300 patients. The mean total score of the questionnaire administered was 143.58 (SD = 18.88). The mean subscale scores for territorial space and personal space invasion were 89.10 (SD = 15.29) and 54.48 (SD = 10.58), respectively. The invasion of territorial space differed significantly between patients with and without children (p = 0.02) and for the number of people living in the residence (p < 0.01).

**Conclusions:**

Attitudes of the nursing staff, such as touching the patient’s possessions without permission and exposing the patient, caused discomfort and violated patient privacy. Patients who were lonelier and had more privacy at home perceived greater invasion of their territorial space by the nursing professionals.

## Introduction

The social meaning of space, i.e., how humans consciously or unconsciously structure their own space and its influences on interpersonal relationships, is studied by proxemics [1], which defines three types of space: fixed feature space (e.g., walls), semi-fixed feature space (e.g., arrangement of furniture, obstacles and decoration), and informal space (e.g., personal territory around an individual’s body) [1–2]. With respect to informal features, every human being have a private space around himself/herself, the size of which depends on the population density of the place where he/she was raised. The space of a person is therefore culturally determined [3] and, regardless of how much a person tries, it is impossible to disregard his/her culture as this determines how an individual perceives the world [2].

The personal space is divided into four distance zones: intimate, personal, social, and public. The distance chosen depends on the relationship between individuals, how they feel, and what they are doing [2]. The intimate zone is reserved for affectively close people that have permission to approach and is the most important for healthcare providers. In the hospital setting, most procedures and interventions are performed at this distance, often without the due affectivity and permission [2–3]. Within this context of the cultural and personal use of space, healthcare providers need to know and respect the limits of the physical distance that should be maintained in different situations of interaction with the patient so that both feel comfortable [1].

By caring for the patient, nurses touch the body and expose it, often without asking permission, adopting an attitude of “power” over the body of the other. Being naked can mean discomfort and embarrassment, feelings demonstrated by expressions of surprise, shame, fear, and nervousness [4]. In the hospital setting, the patient shares his/her space with strangers, other patients and healthcare workers. Consequently, the feeling of space invasion occurs more frequently than in the family environment, since the individuals usually experiences situations of reduced privacy and control over their bodies and the area that surrounds them [5].

Territoriality is the area that individuals claim as their own, defending it from other members of the same species. There are three ways to invade the territory of the patient, invasion by looking, actual invasion when somebody touches the patient’s possessions without permission, and invasion with objects of both the patient's body and the space it occupies [1].

A study conducted in Nepal to evaluate patients’ attitudes towards physical privacy and confidentiality of information during consultation in a public hospital showed that the majority of patients were not comfortable having other patients in the same room. The authors suggest that attention should be given to reorganizing outpatient facilities and that future facilities should provide more privacy [6]. Another international study reported a strategy for working with the issue of patient privacy and satisfaction with healthcare providers and concluded that continued training and education are essential so that healthcare workers remain aware of these issues. The intervention strategies developed to improve patient privacy and satisfaction included the reorganization of the physical space, process management, access control, staff education and training, as well as ethical aspects [7].

The issue in question has an important dimension in the care and should be considered a professional ethical principle. Authors have demonstrated that the violation of personal (staff behavior) and territorial (hospital environment) privacy can threaten the dignity of patients and be misinterpreted by patients, causing constraints or inducing defensive behaviors [8–9], therefore the avoidance of this should be guaranteed by the nursing staff [10]. Accordingly, to ensure clear communication that allows the patient to control decision making [8,11], as well as providing respect, privacy and confidentiality of data are fundamental strategies for the maintenance of the dignity of the patients [11].

The fact that neither the actual invasion nor the perception of invasion is always clearly perceived by healthcare providers or the patient highlights the importance of this study for increasing the awareness of the nursing staff regarding the comprehension of the feelings experienced by patients when their space is invaded. Therefore, the aims of this study were to identify the patient’s perception of invasion of territorial and personal space and to determine whether personal characteristics, housing conditions and characteristics of the hospital unit have an impact on this perception.

## Methods

A cross-sectional, analytical and quantitative study was conducted at a public hospital in the interior of the state of São Paulo, in the internal medicine and maternity wards and in the ward for patients with private health insurance. The internal medicine ward has 22 beds and mainly attends patients undergoing minor surgeries in the hospital. The ward comprises 5 rooms with 2 beds and 2 rooms with 6 beds sharing the same physical space. The maternity ward has 34 beds and attends pregnant and postpartum women and newborns, having 11 rooms with 2 beds and 3 rooms with 6 beds. The ward for private patients has 16 beds divided into 8 rooms with 2 beds each.

The criteria for inclusion in the study were to be aged 18 to 60 years, hospitalized for more than 24 hours and literate. The minimum sample size was calculated, using the calculator available at the website of the *Laboratório de Epidemiologia e Estatística*, *Instituto Dante Pazzanese de Cardiologia*, for a pilot sample of 30 participants. Standard deviation (σ = 30.08) and mean (μ = 126.17) were used to calculate the coefficient of variation (CV = σ/μ; CV = 0.238) and maximum error of the estimate (MEE = CVxσ; MEE = 7.17). The level of significance was pre-established at 5% and the minimum sample size estimated for application of the instrument was 68.

The questionnaire for the characterization of the participants consisted of 12 variables: personal characteristics (gender, age, marital status, education level, and having children), hospital unit (whether or not the patient shared a room in the hospital), and housing conditions (whether or not the patient shared a room, number of people with whom the patient shared the room in their house, which people shared the room, having a personal space at home, number of rooms in the patient’ house, and number of household members).

The Anxiety Due to Territory and Space Intrusion Questionnaire used was designed to identify the feelings of hospitalized patients regarding invasion of their personal and territorial space. The validation study and the cross-cultural adaptation to the Brazilian reality of Anxiety Due to Territory and Space Intrusion Questionnaire was published in 1998 and obtained satisfactory psychometric qualities [5].

The questionnaire consists of 33 questions divided into two subscales, with 19 items in the territorial space invasion subscale and 14 items in the personal space invasion subscale. The response alternatives for each item are measured on a Likert-type scale from 1 (totally unpleasant) to 7 (extremely pleasant), with the total score ranging from 33 to 231. With higher scores indicating greater perception of personal and territorial space invasion [5].

The patients were approached in the hospital and the data collected between January and March 2015. The study was conducted in accordance with national and international ethical guidelines on research involving human subjects and was approved by the Research Ethics Committee of Faculty of Medicine Jundiaí (Authorization No. 859.952/2015).

The data were analyzed through descriptive and inferential analysis using the IBM Statistical Package for the Social Sciences (SPSS, version 20.0). Spearman’s correlation test was used to compare the numerical variables with the scores of the questionnaire. Categorical variables were compared with the scores using the Kruskal-Wallis and Mann-Whitney tests. The error probability adopted in the tests was p<0.05. A trend was considered significant when p≤0.10.

## Results

The sample was composed of 300 patients, with a mean age of 30.9 years (SD = 7.8). There was a predominance of women (n = 279; 93%), married subjects (n = 122; 40.7%), patients with complete high school education (n = 166; 55.5%), and patients who had children (n = 262; 87.3%). The majority of participants were hospitalized in the maternity ward in the rooms with six beds (n = 126; 42%) (Table 1).

**Table 1:** Personal, hospital and housing characteristics of the participants.

Characteristics	n	%
**Marital status**		
Single	58	19.3
Married	122	40.7
Cohabiting	87	29.0
Separated	12	4.0
Divorced	14	4.7
Widowed	7	2.3
**Education level**		
Elementary school	74	24.7
High school	166	55.3
Technical education	40	13.3
Higher education	20	6.7
**Hospital unit**		
Ward for private patients with 2 beds/room	30	10.0
Internal medicine with 6 beds/room	64	21.3
Maternity with 2 beds/room	80	26.7
Maternity with 6 beds/room	126	42.0
**Number of people with whom the patient shares the room in the house**		
None	48	16.0
1 person	191	63.7
2 people	37	12.3
3 people	16	5.3
4 people	8	2.7
**Sharing the room with in the house**		
Father	2	0.7
Mother	5	1.7
Spouse	205	68.3
Child	39	15.3
Siblings	4	1.6
**Number of rooms in the house**		
1 or 2	16	5.3
3	88	29.3
4	133	44.3
5 or 6	63	21
**Number of household members**		
Alone or 1 person	47	15.7
2 people	105	35.0
3 people	91	30.3
More than 4 people	57	19.0

Regarding housing, the majority of participants shared their room (n = 253; 84.7%) with another person (n = 191; 63.7%), which was the spouse (n = 205; 68.3%) and reported not having any personal space in the house (n = 260; 86.7%). The majority of residences had four rooms in the house (n = 133; 44.3%) occupied by two people (n = 105; 35%) (Table 1).

Table 2 shows the total mean score and subscale scores of the responses of the participants regarding invasion of their space. As can be seen in the table, the perception of invasion of territorial space was greater than that of personal space.

**Table 2:** Total score and subscale scores of the Anxiety Due to Territory and Space Intrusion Questionnaire.

	No. of items	Range	Median	Mean	Standard deviation
Invasion of territorial space	19	19–133	91	89.10	15.29
Invasion of personal space	14	14–98	52	54.48	10.58
**Total score**	**33**	**33–231**	**143**	**143.58**	**18.88**

In the territorial space invasion subscale, the three highest means were observed for items 9 (μ = 5.07; SD = 1.38), 5 (μ = 4.86; SD = 1.24) and 10 (μ = 4.85; SD = 1.21). In these the participants reported that touching their personal possessions without permission, changing the bedside table to a position that cannot be reached, and raising or lowering the window blinds without consulting the patient were attitudes of the nursing staff that annoyed them and caused a feeling of invasion (Table 3).

**Table 3:** Situations in which patients reported invasion of their territorial and personal space, according to the Anxiety Due to Territory and Space Intrusion Questionnaire.

	Anxiety Due to Territory and Space Intrusion Questionnaire Items	Median	Mean	Standard deviation
	**Territorial Space Invasion Subscale**			
**1**	The door of your room is closed and a member of the nursing staff walks in without knocking.	5.00	4.13	1.50
**2**	When you are sitting in the chair, the nurse sits on your bed while she is talking.	5.00	4.81	1.33
**3**	The nurse leaves the door open when she leaves your room.	5.00	481	1.29
**4**	The nursing staff speak loudly while working in their ward.	5.00	4.73	1.21
**5**	Your bedside table has been moved to a position that cannot be easily reached by you.	5.00	4.86	1.24
**6**	The nurse takes a chair out of your room without asking if you will use it.	5.00	4.67	1.20
**7**	A member of the nursing staff stumbles into the bed in which you are lying.	5.00	4.68	1.11
**8**	Your bedroom window is closed or opened without asking what you would prefer.	5.00	4.79	1.17
**9**	Without asking your permission, the nurse interferes with your personal belongings in the drawer.	5.00	5.07	1.38
**10**	The blinds in your bedroom window are raised or lowered without asking what you would prefer.	5.00	4.85	1.21
**11**	The nurse enters your room without knocking on the door.	5.00	4.75	1.14
**12**	The cleaners put your personal belongings into the bedside table without asking how you want them arranged.	5.00	4.69	1.16
**13**	The nurse enters your room and begins to change the location of your bed while you are lying down.	4.50	4,56	1.18
**14**	{A member of the nursing staff speaks loudly when talking to you.	5.00	4.62	1.13
**15**	In the time you are resting the cleaners uses the machine to clean the floor.	5.00	4.61	1.19
**16**	The cleaners bang the mop against the foot of your bed while you are lying down.	5.00	4.63	1.11
**17**	In the time you are resting, the nursing staff talk loudly in the hallway.	5.00	4.67	1.16
**18**	The nurse leaves the door and the windows of your room open and the wind blows on your body.	4.50	4,62	1.16
**19**	During the night, while you are asleep, the nurse turns on the light in your room to take care of the next patient.	4.00	4.59	1.04
	**Personal Space Invasion Subscale**			
**1**	You lie in bed. The nurse leans over to arrange you in the bed and you feel her breath against your face as she speaks.	5.00	4.13	1.50
**2**	The nurse stands near the head of your bed when talking to you.	2.00	3.03	1.64
**3**	You are sitting in the chair. The nurse comes over and puts a hand on your shoulder as she talks.	2.00	2.86	1.47
**4**	A member of the medical team sits next to your bed while talking to you.	2.00	2.83	1.50
**5**	The nurse holds your hand for a few minutes after placing the thermometer under your arm.	3.00	3.03	1.47
**6**	After asking you some questions, a member of the medical team begins to examine different parts of your body.	5.00	4.21	1.16
**7**	The nurse performs a technical nursing procedure in a more intimate area of your body.	5.00	4.24	1.24
**8**	The nurse holds your hand while discussing what activities will be developed with you during the day.	3.00	3.05	1.50
**9**	You are lying in bed. The nurse leans over you to clean your bed.	5.00	4.30	1.34
**10**	{A member of the medical team holds your hands while you are talking about a problem.	2.00	3.02	1.54
**11**	A member of the medical team holds your hands while you are talking about a problem.	3.00	3.15	1.65
**12**	The nurse changes your clothes without putting up the screen.	6.00	6.13	1.21
**13**	The nurse performs a technical nursing procedure in a more intimate area of your body without putting up the screen.	6.00	6.17	1.20
**14**	The medical team gathers around your bed and discusses your illness.	4.00	4.10	1.79

In the personal space invasion subscale, the three highest means were found for items 13 (μ = 6.17; SD = 1.20) and 12 (μ = 6,13; SD = 1.21). These showed that embarrassing attitudes occur when the nursing staff conduct a technical procedure in an intimate area or change the patient's clothes without a screen (Table 3).

Table 4 shows the comparison of invasion of territorial and personal space with the other variables studied. The statistically significant differences indicate that patients who no had children (p = 0.02) and those living with only one people in the residence (p < 0.01) perceived greater invasion of their territorial space. The significant trends indicate that patients who shared the room (p = 0.09) or were hospitalized in the maternity ward (p = 0.10) felt less personal space invasion.

**Table 4:** Comparison of sample characteristics with the subscale scores of the Anxiety Due to Territory and Space Intrusion Questionnaire.

	Invasion of territorial space				Invasion of personal space			
	Median	Mean	SD	P-value	Median	Mean	SD	P-value
Sex*				0.13				0.21
Female	90.00	88.79	15.38		52.00	54.70	10.53	
Male	96.00	93.33	13.67		52.00	51.52	10.95	
**Marital status**				0.69				0.38
Single	92.00	91.72	17.29		51.50	55.14	11.18	
Married	90.50	88.41	14.92		53.00	54.34	10.09	
Cohabiting	88.00	88.25	14.12		53.00	55.48	10.64	
Separated, Divorced or Widowed	91.00	89.33	16.06		51.00	51.21	10.69	
**Education level**				0.19				0.28
Elementary school	91.00	89.54	13.80		51.50	53.23	10.67	
High school	87.00	88.09	16.38		52.00	54.64	10.57	
Technical or Higher education	95.00	91.38	13.80		55.00	55.56	10.52	
Children*				**0.02**				0.84
Yes	89.00	88.25	14.73		52.50	54.33	10.35	
No	96.50	95.00	17.84		51.50	55.50	12.16	
**If children, number**				0.89				0.54
1	89.50	88.63	14.01		53.00	54.41	10.14	
2	87.50	88.00	16.33		51.50	53.34	10.82	
3 or more	91.00	87.26	14.84		54.00	55.64	10.39	
**Shared room**				**0.09**				0.70
No (sleep alone)	94.50	93.17	17.24		52.50	54.23	11.29	
Yes	89.50	88.33	14.79		52.00	54.53	10.46	
**Number of other people that share the room**				0.15				0.80
None	94.50	93.17	17.27		52.50	54.23	11.28	
1 or 2 people	90.50	88.71	14.78		52.00	54.56	10.00	
3 or 4 people	86.00	84.79	14.78		50.50	54.21	14.41	
Private Space in the house*				0.30				0.63
Yes	96.50	90.67	19.87		53.50	55.20	12.44	
No	91.00	88.83	14.51		52.00	54.30	10.24	
**Number of rooms in the house**				0.13				0.78
≤3	93.50	92.05	14.51		52.00	54.53	11.03	
4	86.00	87.47	14.67		53.00	54.96	10.61	
≥5	92.00	87.71	17.23		52.00	53.38	9.81	
**Number of people living in the residence**				**0.01**				0.75
≤1	97.00	97.46	15.44		51.00	53.70	12.19	
2	86.00	87.56	13.41		52.00	53.98	9.84	
3	84.00	86.78	15.81		53.00	55.27	10.44	
≥4	95.00	88.77	15.64		53.00	54.77	10.89	
**Hospital unit**								
Ward for private patients with 2 beds/room	94.00	89.34	15.21	**0.10**	54.00	55.55	11.21	0.26
Internal medicine with 6 beds/room	94.50	92.25	15.56		52.50	53.89	11.22	
Maternity with 2 beds/room	92.00	90.61	15.59		56.00	56.08	10.19	
Maternity with 6 beds/room	86.00	86.68	14.66		52.00	53.56	10.35	

Kruskal-Wallis Test/ *Mann-Whitney Test.

SD: Standard deviation.

The correlations between age and Invasion of territorial space (p = 0.14) and Invasion of personal space (p = 0.50) were not statistically significant.

## Discussion

The greater perception of territorial invasion is probably due to the fact that patients are somehow prepared for personal invasion in the hospital as they are aware that the approximation by unknown people to perform procedures and to touch their body is part of the treatment. However, territorial invasion is less tolerated since the instinctive drive is stronger, directing the control to personal possessions. Territorial invasion could have been unconsciously interpreted by the patients as a threat due to their vulnerable and dependent condition. In fact, other authors [9] have reached a similar conclusion regarding the more frequent occurrence of work activities in the patients’ room.

International studies [6–7] have demonstrated the same problems of invasion by healthcare providers as those raised in Brazilian studies and in the present one. Touching the patient’s possessions without permission, changing the bed side table to a position that cannot be reached, and raising or lowering the window blinds without consulting the patient are attitudes of the nursing staff that cause much discomfort. Healthcare providers need to be more attentive to the patient’s space and respect the territoriality established by them, often with their personal objects and possessions. Small actions, such as changing the place of the cell phone or slippers, can symbolize the removal of territory and generate strong feelings of discomfort [1].

Physical exposure of the patient is another factor that needs to be highlighted. Performing a technical procedure in an intimate area and changing the patient’s clothes without a screen are actions that cannot be accepted and that must be constantly supervised and addressed by the team, as privacy is a necessity and right of every human being and is essential to the maintenance of dignity. Embarrassment due to exposure of the body, lack of intimacy and disrespectful behavior by nursing professionals has also been reported by patients in other studies [12–13].

In a study with 40 patients, using the same scale, three of the four situations identified by the authors that received the highest mean scores were the same as those of the present study and were even scored higher by the patients: “the nursing staff touch the patient’s possessions in the drawer without his/her permission”, “the nursing staff change the patient’s clothes without closing the screen”, and “the nursing staff perform a technical procedure in an intimate area without closing the screen” [12].

Embarrassment of the patient in the hospital environment is generally caused by exposure of the body to other patients, relatives and healthcare workers. Nudity in front of strangers can be deeply iatrogenic. Within this context, the age, gender and culture of the affected subjects can directly affect the communication dynamics. The results found corroborate studies in the literature in which the authors observed discomfort of the patient with nudity and body exposure and manipulation [6–7,14–15]. The patients reported that requesting permission to manipulate their body, to examine them or to perform other care/procedure shows consideration and attention on the part of the professional, which makes the patient feel valued and in control of the situation. This approach may minimize the effects of the invasion and the feeling of being seen as an object [14].

The respect of territory and personal space represents an ethical and respectful approach to patients, which can permit to maintain their dignity even under vulnerable conditions, favouring their recovery, as most studies have highlighted [7–11].

The results of the present study are in line with those of other national and international studies, which have investigated similar problems concerning patients’ perception of invasion of their territory and personal space by heath care providers [6–8,10–11]. Other authors [15] suggest that unnecessary actions and exposure responsible for discomfort of patient should be avoided, because potentially detrimental to individual dignity and treatment results.

The limitations of this study that can be mentioned include the non-random selection of the participants, the fact that it was performed in only one public hospital in Brazil, which serves predominantly the maternal and child public and, consequently, the significant number of female participants, unbalancing the sample with respect to gender. Other limitations need be reported. The cross-sectional nature of our study can only provide associations, the study evaluated only self-reported perceptions of patients and not actual practice by healthcare staff and the sample is not representative of other settings in the country.

Further studies involving other health professionals and institutions, with sizes and characteristics different to the study hospital, should be carried out with the purpose of sensitizing and identifying what actions are being implemented in order to guarantee the privacy, autonomy and information required to guarantee the dignity of patients.

## Conclusions

The patients felt their space invaded in the hospital environment; with this perception of invasion being greater regarding the territorial space than the personal space.

The findings of this study, by self-reported perception of patients, showed that the attitudes of the nursing staff, such as touching the patient’s possessions without permission and exposing the patient, caused discomfort and violated patient privacy. Patients who were lonelier and had more privacy at home perceived greater invasion of their territorial space by nursing professionals.

Healthcare should respect the individuality and dignity of the patient, not only including changes in the physical space, but also in the actions and behavior of healthcare providers regarding patient privacy.
